# Validation of Fabric-Based Thigh-Wearable EMG Sensors and Oximetry for Monitoring Quadriceps Activity during Strength and Endurance Exercises

**DOI:** 10.3390/s20174664

**Published:** 2020-08-19

**Authors:** Riccardo Di Giminiani, Marco Cardinale, Marco Ferrari, Valentina Quaresima

**Affiliations:** 1 Department of Biotechnological and Applied Clinical Sciences, University of L’Aquila, 67100 L’Aquila, Italy; 2 Aspetar Orthopaedic and Sports Medicine Hospital, Doha PO Box 29222, Qatar; Marco.Cardinale@aspetar.com; 3 Department of Computer Science and Institute of Sport, Exercise and Health, University College London, London WC1E 6BS, UK; 4 Department of Life, Health and Environmental Sciences, University of L’Aquila, 67100 L’Aquila, Italy; marco.ferrari@univaq.it (M.F.); valentina.quaresima@univaq.it (V.Q.)

**Keywords:** wearable biosensors, NIRS, EMG, oximetry, skeletal muscle function, quadriceps

## Abstract

Muscle oximetry based on near-infrared spectroscopy (NIRS) and electromyography (EMG) techniques in adherent clothing might be used to monitor the muscular activity of selected muscle groups while exercising. The fusion of these wearable technologies in sporting garments can allow the objective assessment of the quality and the quantity of the muscle activity as well as the continuous monitoring of exercise programs. Several prototypes integrating EMG and NIRS have been developed previously; however, most devices presented the limitations of not measuring regional muscle oxyhemoglobin saturation and did not embed textile sensors for EMG. The purpose of this study was to compare regional muscle oxyhemoglobin saturation and surface EMG data, measured under resting and dynamic conditions (treadmill run and strength exercises) by a recently developed wearable integrated quadriceps muscle oximetry/EMG system adopting smart textiles for EMG, with those obtained by using two “gold standard” commercial instrumentations for EMG and muscle oximetry. The validity and agreement between the wearable integrated muscle oximetry/EMG system and the “gold standard” instrumentations were assessed by using the Bland-Altman agreement plots to determine the bias. The results support the validity of the data provided by the wearable electronic garment developed purposely for the quadriceps muscle group and suggest the potential of using such device to measure strength and endurance exercises in vivo in various populations.

## 1. Introduction

The recent progress in wireless technologies and various types of wearable sensors has recently been comprehensively reviewed [1]. Furthermore, the applications of wearable technology for assessing the biomechanical/physiological parameters of the athlete and the use of training-monitoring technology have recently been discussed in depth by Cardinale and Varley [2] and Seshadri et al. [3]. There is nowadays supporting evidence that using technology to measure and quantify human responses to exercise in vivo has merit in improving our understanding of the effects of exercise regimes as well as providing more information to personalize training interventions. With the evolution of micro technologies, the best solution would be to embed sensors in wearable garments.

The main types of smart wearables and the most relevant subsystems of a smart garment, as well as their communications architecture have been recently described [4]. Park et al. [1] illustrated the advantages of a wearable optical sensor. The typical use of such sensors is the measurement of oxygen saturation (SpO_2_) by pulse oximetry. This technique uses visible and near-infrared (NIR) light emitting diodes (LEDs) and it is a suitable aid for clinical decision making and a valuable tool in triaging potentially hypoxic patients. This microtechnology can be implemented either at home or in general practice surgeries/assessment centers, because pulse oximetry helps medical doctors in determining which patients require further assessment or treatment [5]. For example, with the increase in COVID-19 cases, such technology has been implemented to determine the deterioration of the respiratory symptoms and help with COVID -related diagnosis [6].

Therefore, the use of wearable biosensors, which allow the continuous and non-invasive measurement of physiological signals, can be fundamental for an improved monitoring of active lifestyles and for the diagnosis/treatment of specific pathological conditions. In particular, the possibility of assessing muscle activity not only in terms of activation but also in terms of oxygen availability in vivo represents a significant advancement to optimize any training and rehabilitation exercise prescription.

It is well known that the energy for muscle work mainly comes from the oxidation of glucose and lipids in muscle fibers. Therefore, optoelectronic devices, driven by NIRS, can play a substantial role in investigating muscle oxidative metabolism at rest and during static or dynamic exercise modalities. In fact, muscle NIRS oximetry, based on the oxygen dependent characteristics of NIR light [7], can provide non-invasively information about the changes in the oxygenation state and hemodynamics in skeletal muscle tissue. The NIRS signals are the result of the weighted average of the oxygen saturations of the heme groups of hemoglobin (Hb) in the arterioles, capillaries, and venules as well as the heme group of myoglobin (Mb) in the muscle cells cytoplasm. Specifically, the NIRS oximeters provide a continuous measure of regional oxyhemoglobin (O_2_Hb) saturation on a scale from 0 to 100%; this parameter reflects the balance between oxygen supply and oxygen demand mainly at the superficial level of muscles [8]. In addition, regional O_2_Hb saturation represents the tissue reserve capacity following tissue oxygen extraction. Muscle oximetry has been successfully used both in applied sports contexts and in different fields of medicine [8,9]. Historically, the first commercial portable wireless muscle oximeter was released in 2006 by Artinis Medical System (Einsteinweg, Netherlands). In contrast, electromyography (EMG) is a technique that is already well established, having already been used for more than 100 years to record electrical muscle activity.

The first study using the simultaneous recordings of EMG and NIRS was presented in the literature almost twenty years ago [10], suggesting the potential for such techniques to better describe muscle demands during exercise. A recent review has nicely summarized the role of the EMG-NIRS combination in clinical practice [11]. Recently, several prototypes integrating EMG and NIRS have been developed [12,13,14,15,16]. However, those devices presented several limitations; in particular, they did not measure regional muscle O_2_Hb saturation (instead they provided only oxygenation changes) and partially used full textile sensors for EMG. Textile electrodes embedded in clothing have been suggested as a practical alternative to traditional surface electromyography for assessing muscle excitation during functional movements [17,18]. The feasibility of successfully using a new wearable integrated NIRS-oximetry/EMG system (wiNIREM), adopting smart textiles developed by Ohmatex ApS (Denmark), to monitor dynamically exercising leg muscle groups has already been demonstrated by our group [19]. To the best of our knowledge this was the first proof of concept of the integrated wiNIREM wearable textile device for assessing exercise activities in vivo and quantifying muscle exertion.

Considering the potential for such garments to be used for clinical and sporting purposes, we aimed to compare the regional muscle O_2_Hb saturation and surface EMG data, measured under resting and dynamic conditions by wiNIREM, with those obtained by using the two “gold standard” commercial instrumentations for EMG and muscle oximetry. The results of this study support the reliability of the data provided by the combined fabric-based wearable EMG sensors and tissue oximetry purposely designed to monitor strength and endurance exercises.

## 2. Materials and Methods

### 2.1. Participants

Five male sport science students (age: 23.2 (1.4) years; body mass: 75 (1.3) kg; stature: 177.8 (2.4) cm; body mass index (BMI): 23.8 (0.5) kg/m^2^) voluntarily participated in the present investigation, giving written informed consent, and the experiments were conducted in the University of L’Aquila according to the World Medical Association Declaration of Helsinki. The Internal Review Board approved the study (protocol number 23/2017; https://univaq.it/en/section.php?id=1527). Inclusion criteria were: normal BMI (18–25 kg/m^2^), subjects should be experienced in endurance exercises (i.e., running on treadmill at speeds ranges between 4 and 16 km/h) and strength exercises performed on a Smith Machine (i.e., maximal isometric voluntary contraction during high squat and isotonic-dynamic high squat). The exclusion criteria were smoking, peripheral vascular disease, history of back pain, musculoskeletal and/or tendinous injuries and severely delayed muscle soreness in the lower extremities.

### 2.2. Wearable Integrated EMG Sensors and Oximetry

**Figure 1:**
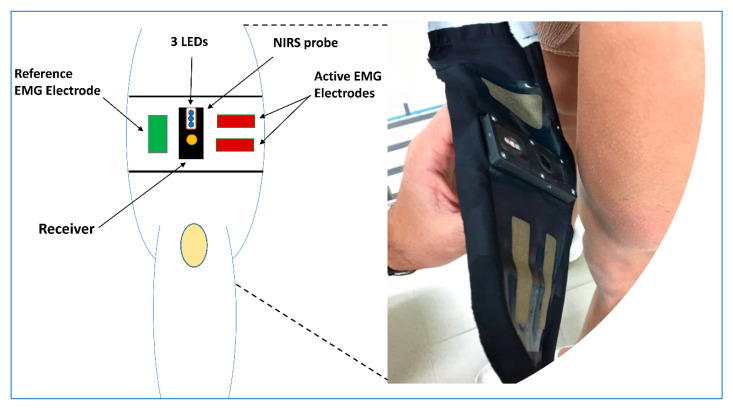
Details of the integrated system (wiNIREM) developed by Ohmatex ApS, including the fabric-based thigh wearable EMG sensors and the NIRS-oximetry probe. Scale 1:4.

The wearable integrated garment developed by Ohmatex ApS is referred to as wiNIREM (Figure 1). Ohmatex ApS (Skanderborgvej 234, 8260 Viby J, Denmark; http://www.ohmatex.dk/) is a Danish company specialized in the development of intelligent textile devices and surveillance gear, including those for medical, sports and space applications. In 2014, Ohmatex ApS was commissioned by the European Space Agency (ESA) (ESA Contract No. 4000111950) to design and integrate NIRS-oximetry and surface EMG techniques in adherent clothing with the aim to monitor the muscular activity of astronauts during lower limb exercises and at rest condition. The garment was designed to be resistant to wear, tear and sweat (waterproof); the structure was assembled to provide uniform placement of the devices and electrodes on the quadriceps and to avoid motion artefacts.

#### wiNIREM

The NIRS-oximetry and EMG electronics of the wiNIREM were mounted on either side of the fabric of the test sleeve. An elastomeric film designed for apparel (Bemis Associates Inc., Shirley, USA) was laminated to the surface of the sleeve fabric to protect the electronics housing from moisture seeping through the fabric and to protect the cables to the electrodes from damage. The textile EMG electrodes were constructed using a commercially available silver coated fabric designed for medical use (Shieldex Technik Tex, Statex, Bremen, Germany) previously used in studies evaluating textile electrodes for EEG and EMG signal detection [20,21]. The silver-coated fabric was tested for skin compatibility/safety (DIN EN ISO 10993-5). The textile electrodes interfaced with EMG electronics through openings in the sleeve, the elastomeric film, and the EMG housing. Active electrodes (6 × 2 cm in size) were placed in the sleeve positioned parallel each other with a 2-cm distance between them (Figure 1). A reference electrode of 6 × 3 cm was placed away from the active recording site. EMG was amplified with a gain of 10,000 with a passband filter of 20–350 Hz and sampled at 2000 Hz, full wave root mean square (RMS, 100 Hz), Common Mode Rejection Rate (CMRR): 112dB. A bespoke NIRS-oximetry probe weighing 34 g was developed to be embedded in the wearable solution. The LEDs, each transmitting at two wavelengths (760 and 850 nm), were positioned at 3, 3.5, and 4 cm from a high-sensitivity three pairs photodetector receiver in a spatially resolved spectroscopy (SRS) configuration and embedded between the EMG active electrodes and ground (Figure 1). The LEDs and the receiver were covered by a clear polycarbonate cover which acts as a mechanical barrier to direct skin contact and ensures compliance with requirements to touch temperature. This device simultaneously uses the modified Beer–Lambert law and SRS methods with a proprietary algorithm to calculate the concentration changes of tissue oxy(+myo) hemoglobin, (O_2_Hb), deoxyhemo(+myo)globin (HHb) and total hemo(+myo)globin (tHb). Therefore, tissue oxygen index (TOI) is expressed in percentages (%) and calculated as in Equation (1):

(1) $$TOI\;=\;[O_{2}Hb]/\left(\left[O_{2}Hb\right]+\left[HHb\right]\right)\times 100$$

which is the ratio of O_2_Hb to tHb. TOI reflects the dynamic balance between oxygen supply and consumption [8]. During all testing, the system was connected to a personal computer for data acquisition (10 Hz), and analogue-to-digital conversion and subsequent data analysis. The NIRS-oximetry and EMG sub-systems of the wiNIREM were synchronized using a bespoke software solution (Smart-Collect, Ohmatex, Denmark) and connected to a PC and medically approved power supply to guarantee safety of the subjects. The board has an analog-to-digital converter of 12-bit. The wiNIREM safety was evaluated by an independent company (Danish Aerospace Company, Odense, Denmark).

### 2.3. Experimental Protocol

A single-group, crossover study design with repeated measures was used. The following procedure was repeated twice: the wiNIREM was positioned on the left thigh and the “gold standard” instrumentation on the right thigh (PortaMon-Artinis, Einsteinweg, The Netherlands; MuscleLab-Ergotest Innovation, Porsgrunn, Norway); and gold standards were positioned on the left thigh and wiNIREM on the right thigh. Thus, the comparisons between the signals from the gold standards and wiNIREM were performed during the same type of exercise from different legs (right vs. left), or from the same leg and type of exercise but in different lab visits.

**Figure 2:**
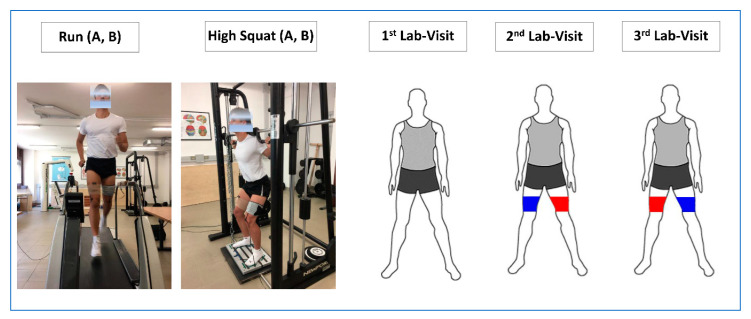
Design of the study. 1st Lab-Visit: participants familiarized themselves with the experimental procedures. 2nd Lab-Visit: Session A: participants performed the maximal isometric voluntary contractions (MIVCs) during High Squat (A), dynamic High Squat (A) and run (A). The positioning of wiNIREM on one leg and the gold standard instrument on the other leg was random. 3rd Lab-Visit: Session B: participants performed MIVCs (B), dynamic High Squat (B), and Run (B). The positions of the wiNIREM and the “gold standard” instrumentations were swapped with respect to those in Session A.

Each participant visited the Laboratory three times separated by at least one day of recovery, and the repeated measurements were carried out at same time of day in order to avoid circadian effects (Figure 2). Participants performed the testing in two separate sessions (treadmill run and strength tests). During Testing Session A, wiNIREM was positioned on one leg and the gold standards on the other leg; conversely, during Testing Session B, the positions of the wiNIREM and the gold standards were swapped. The positioning of wiNIREM on one leg and the gold standards on the opposite leg was random in Testing Session A and this same order was maintained for Testing Session B. The comparison of wiNIREM versus gold standards was performed on the dominant leg (Figure 2). To prepare the subjects for the exercise tasks and reduce the risk of injury, before starting the exercises, the subjects performed a standardized warm-up consisting of an 8-min run on a treadmill at 5 km/h, 2-min dynamic stretching and 5-min leaps in mono and bipedal stance. This activity also offered the opportunity to check the quality of the data collected in real time before starting the experimental trials.

Following the warm-up, the subjects were asked to perform the following exercises (Figure 2):

- A 3-min submaximal treadmill run at 4, 10, 12, 14 and 16 km/h on a motorized treadmill. A 5-min pause was adopted between trials. The trials were performed in a random order. Several phases were marked in the EMG/NIRS signals: rest (1-min), incremental, to reach the speed (1 km/h per s), during the duration of the trial at the selected speed (3-min), and during the recovery phase (1-min).
- Maximal isometric voluntary contraction (MIVC) was performed while pushing against a barbell locked in a Smith machine. During the execution of this task, the participants assumed a high squat position (knees flexed at about 120 degrees) before starting the maximal isometric task. Once in position, participants were given a verbal command to push as hard as possible against the barbell for 6 s. They performed three trials (6 s each) with a 3-min pause between them. The highest value was used for analysis.
- Dynamic high squat exercise. This task consisted of ten isotonic actions (alternating eccentric-concentric action) with a submaximal load equal to 50% of the MIVC measured in the previous task. The subject, from a standing position, flexed the knees up to 120 degrees and back near to full extension for a total of eight repetitions on a Smith machine. An electronic metronome was used to standardize the speed of concentric and eccentric actions. The subjects followed a rhythm of 1 Hz. The recording of the EMG/NIRS signals began when the participant assumed a standing position (1-min rest), then the participant flexed the knee to reach the high squat position and lifted the barbell to the starting point on the Smith Machine (standing position with the barbell on the shoulder-positioning phase), then the participant started the dynamic high squat (eight repetitions) lowering (eccentric contraction) and lifting (concentric contraction) the barbell. Once the last repetition was performed, the barbell was placed on the support and the signals were recorded for a 1-min (recovery phase).

### 2.4. Sensor Positioning

**Figure 3:**
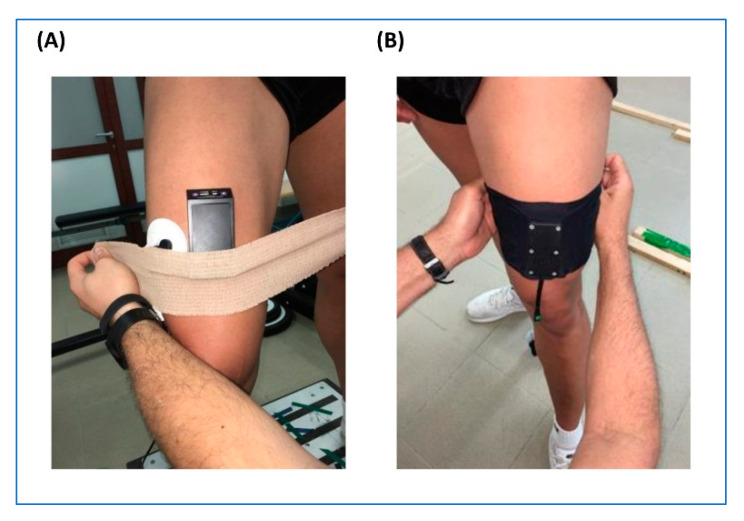
Sensor positioning. (**A**): PortaMon and MuscleLab; (**B**): wiNIREM.

The wearable sleeve was worn and fixed by a Velcro brand fastener adapting it to the individual quadricep size and the shape of the leg. The two active EMG electrodes of the wiNIREM were positioned on the muscle belly of vastus lateralis, while the NIRS probe and the reference EMG electrode were firmly placed over the rectus femoris and vastus medialis, respectively (Figure 3). The EMG textile required the pre-treatment with saline solution to make sure electrodes contact without conductive gel. The devices used for validation of the NIRS-oximetry and EMG signals were the PortaMon and the Muscle Lab, respectively. Both are considered “gold standards” for assessing NIRS-oximetry and EMG related parameters, because they have been validated and have been extensively used in basic research and clinics [9,22].

The EMG recording of the Muscle Lab, currently in use at the European Astronaut Centre [23], was filtered with a fourth-order Butterworth bandpass filter (8–600 Hz). Finally, a hardware circuit network converted the filtered EMG signals (frequency response of 0–600 kHz, averaging constant of 100 ms, and total error of ±0.5%). The root mean square (RMS) signal was then sampled at 100 Hz with a 16-bit A/D converter (AD637 o AD536 circuit (Analog Devices Inc., Norwood, MA, USA).The EMG was detected by using triode electrodes (T3402M, nickel-plated brass, electrode diameter = 1 cm, interelectrode distance = 2 cm, Thought Technology Ltd., Montreal, Canada). The electrodes were fixed longitudinally over the muscle belly according to the recommendations of SENIAM [24]. Prior to gel-coated electrode placement, the skin was shaved and cleaned with alcohol to minimize impedance (<5 kΩ). To prevent motion artefact, cables were secured using elastic bands.

The PortaMon is a compact and light (8.3 × 5.2 × 2 cm; 75 g) muscle oximeter. It is a dual-wavelength (760 and 850 nm), continuous wave system, containing three pairs of LEDs in an SRS configuration with a source–detector spacing of 3, 3.5, and 4 cm. The instrument uses the modified Beer–Lambert law and SRS methods to calculate O_2_Hb saturation as the WINIREM. PortaMon is connected to a personal computer via Bluetooth for data acquisition at 10 Hz.

### 2.5. Statistics

Statistical analysis was conducted by using SigmaPlot 14 (Systat Software, Inc, San Jose, CA, USA). The validity and the agreement between the wiNIREM and the “gold standard” instrumentation were assessed by using Bland–Altman agreement plots [25]. This is deemed the best statistical approach; it is also the most widely used method for validating measurements. We established that the differences within ±2 standard deviations (SD) are not practically or clinically important, and that the 2SD is considered to be the “limit of agreement”, indicating that the two methods can be used with similar accuracy to measure EMG_RMS_ and TOI. The agreement/bias (between the two methods of measurement of the TOI signal), was calculated as the mean absolute difference (bias ±1.96 SD [95% Confidence Interval- Limits of Agreement, LOA]) in resting and exercising TOI%. The difference between devices was calculated as the mean TOI% of PortaMon minus wiNIREM. The same approach was used to assess the accuracy of the EMG signals.

## 3. Results and Discussion

### 3.1. wiNIREM versus PortaMon

**Figure 4:**
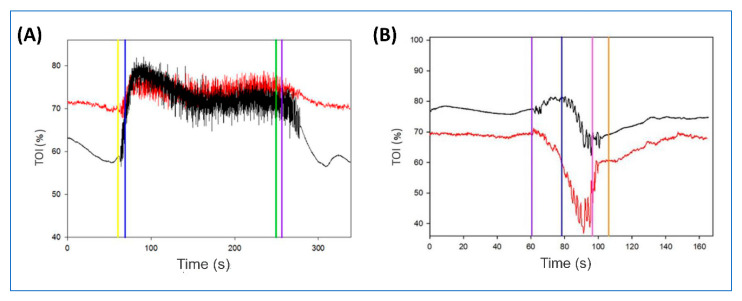
Representative example of the tissue oxygenation index (TOI, %), measured on the right (wiNIREM, red line) and the left (PortaMon, black line) rectus femoris during a 10-km/h running (**A**), where yellow vertical line: incremental; light blue: start; green: stop; violet: recovery, and dynamic high squat (**B**), where violet vertical line: positioning; light blue: start; pink: stop; orange: recovery. For the details see the “Material and Methods” section.

**Figure 5:**
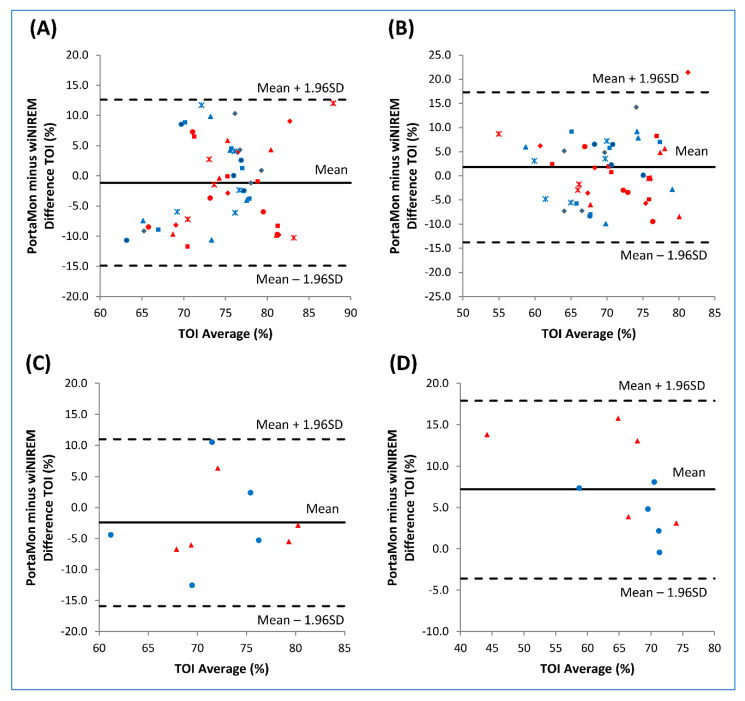
Bland–Altman plots of the difference of rectus femoris (RF) tissue oxygenation index (TOI, %) provided by the two oximeters (wiNIREM and PortaMon) during run (upper panels; **A**,**B**) and dynamic high squat performed at 50% of maximal isometric voluntary contraction (lower panels; **C**,**D**). The comparisons were performed on both legs (red: right RF; blue: left RF). *Upper panels:* each point was obtained averaging the TOI values of the last 20 s at rest (**A**) and the last 20 s of submaximal treadmill run at several speeds (**B**). Triangle: 4 km/h; circle: 10 km/h; square: 12 km/h; rhombus: 14 km/h; asterisk: 16 km/h. *Lower panels*: each point was obtained in the last repetition at rest (**C**) and during dynamic high squat performance (**D**). The upper and lower dotted horizontal lines correspond to ±1.96SD limits of agreement and the center solid line represents the bias.

TOI signals showed similar quality during running and dynamic high squat (Figure 4). Specifically, the TOI measured with wiNIREM and PortaMon overlap during running (Figure 4A), whereas during dynamic high squat the values measured with the PortaMon tended to be higher than those recorded with wiNIREM although the pattern was the same (Figure 4B). In the treadmill run, the mean bias (± standard deviation, SD) measured during running at different speeds ranged from −0.3 to 1.7 with SD from 5.1 to 7.4. The mean bias at each speed was 4 km/h = 0.6 ± 7.0 (LOA = −13.2–14.4); 10 km/h = −0.3 ± 5.3 (LOA = −11.7–11.1); 12 km/h = 1.7 ± 6.1 (LOA = −10.3–13.7); 14 km/h = 0.9 ± 7.4 (LOA = −15.6–21.6); 16 km/h = 0.8 ± 5.1 (LOA = −17.5–25.3), respectively. When all speeds were pooled, mean bias was −1.2 ± 7.0 (LOA = −14.9–12.6) at rest (Figure 5A) and 1.8 ± 6.1 (LOA = −13.8–17.3) during running (Figure 5B). In the dynamic high squat, mean bias was −2.4 ± 6.9 (LOA = −15.9–11.0) at rest (Figure 5C) and 7.2 ± 5.5 (LOA = −3.6–17.9) during dynamic high squat (Figure 5D).

These values are smaller than those reported by other authors when comparing the PortaMon to another muscle NIRS-oximeter (MOXY, Fortiori Design LLC, Hutchinson, Minnesota, USA) [26]. In the latter study, the comparisons between the two muscle oximeters were performed during isometric contractions of the quadriceps muscle using an isokinetic leg-extension task, and during an incremental arm-crank exercise positioning the oximeters on the biceps brachii. In the present investigation, the comparisons between devices were performed with signals recorded during running (from 4 to 16 km/h) and during dynamic high squat (isotonic contractions characterized by stretch-shorten cycles). These exercises, characterized by large transient ground reaction forces at impact (i.e., running) and wide range of motion (i.e., dynamic high squat), could induce motion artefacts between the skin and the sensors placed on the rectus femoris, which in turn could affect the quality of the signals. In this validation study, the oximeter embedded in the textile structure was assembled and designed to avoid motion artefacts and the results show the validity of the textile design to retain the sensor in place.

### 3.2. wiNIREM versus Muscle Lab

**Figure 6:**
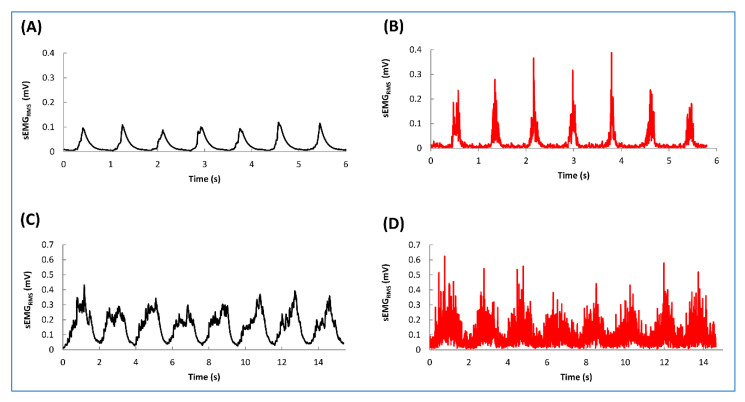
Representative example of the surface electromyographic activity (sEMG_RMS_) recorded on the right (wiNIREM, **B**,**D**) and the left (Muscle Lab, **A**,**C**) vastus lateralis (VL) during an epoch (seven foot contacts) of submaximal treadmill run at 10 km/h (**A**,**B**) and during an eight repetitions of dynamic high squat (**C**,**D**). The running patterns (contact and aerial phases) can be easily identified. For the details see the “Material and Methods” section.

**Figure 7:**
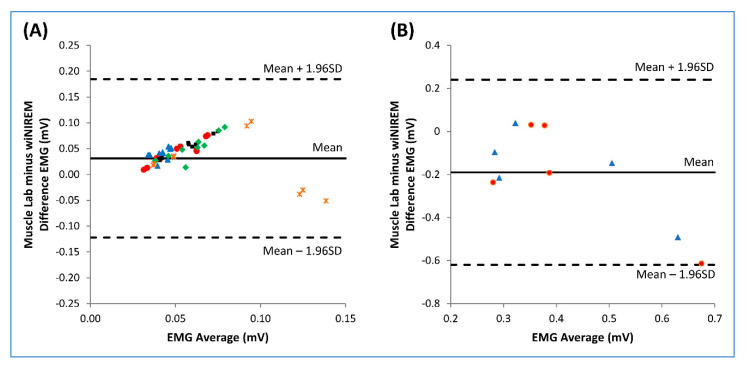
Bland–Altman plots of the difference of vastus lateralis (VL) surface electromyographic activity (sEMG, Root Mean Square-RMS) obtained by wiNIREM and Muscle Lab during run (**A**) and dynamic high squat (**B**). (**A**) Each point was obtained with the RMS values of the last 20 s of submaximal treadmill run performed at each speed (blue triangle = 4 km/h, red circle = 10 km/h, black square = 12 km/h, green rhombus = 14 km/h and orange asterisk = 16 km/h). (**B**) Each point was obtained with the RMS values corresponding to the first (blue triangle) and last repetition (red circle) of the dynamic high squat performed at 50% of maximal isometric voluntary contraction. The comparisons were performed on the dominant leg. The upper and lower dotted horizontal lines correspond to ±1.96SD and the center line represents the bias.

The EMG activity is clearly identifiable and similar between the devices giving confidence about its ability to be used in the field (Figure 6). The amplitude of the wiNIREM EMG textile was higher than the Muscle Lab in both the running and the dynamic high squat tasks (Figure 6). It is plausible that the main difference is related to the signal processing of the two units, but motion artefacts (for example the textile moving relative to the skin during dynamic exercises), could also explain some of the observed differences. The mean bias (±SD) was 0.03 ± 0.08 (LOA = −0.12–0.18) for the runs (Figure 7A) and −0.21 ± 0.22 (LOA = −0.62–0.24) for the dynamic high squat (Figure 7B). The values reported are similar to a previous study that compared textile electrodes vs. conventional EMG systems [18] suggesting that the magnitude of muscle activity can be determined with the current textile configuration during dynamic exercise. Considering the differences in electrode configuration, sampling and cable characteristics, filtering algorithms, and muscle surface areas covered between the two devices used, the bias shown is acceptable and indicates that the wiNIREM device, when properly prepared and worn, can provide valid information about muscle function “in vivo” during typical strength and endurance exercises.

### 3.3. wiNIREM

The wiNIREM and its integrated software showed an accuracy similar to the NIRS-oximetry and EMG instrumentations currently available on the market, and appears suitable for research and training monitoring being the selected exercises some of the fundamental movements in several sport activities and athlete performances. The integrated system compares favorably with the reference/gold standard devices used for comparisons when average values of discrete epochs are used for analysis/comparisons. The level of acceptable agreement for both methods is within the limits of agreement and present values are better than the ones reported in previous studies comparing NIRS-oximetry devices [26] and EMG devices [17,18].

### 3.4. wiNIREM: Practical Applications

The EMG textile and NIRS signals of the wiNIREM were found to be able to detect the squeezing effect due to muscle contraction on the blood vessels during the run at impact phase on the ground [19]. In fact, EMG and NIRS signals appear out of phase; in other words, the muscle activation during heel strike occurs before the increase in TOI. Interestingly, these neuromuscular and metabolic patterns are cyclical over time when running. Therefore, the two integrated signals might be used to assess and monitor specific physiological characteristics of an individual, such as the anaerobic threshold. The access to real-time information on physiological responses to exercise could be used to optimize the endurance performance in real time (i.e., a race) and/or modify exercise prescriptions (e.g., duration of recovery and intensity of activities), as well as to assess performance in vivo. Similarly, during dynamic high squat, the wiNIREM reveals a typical integrated pattern in which muscle activation is constant (i.e., the amplitude of EMG_RMS_, the power of the signal), but it is accompanied by a decrease in TOI over the repetitions.

In our previous study implementing this technology, we observed median frequency alterations of the EMG signal in combination with the TOI decrease during exhaustive isometric contractions [19] being able to describe in real time the increased neuromuscular and metabolic needs for sustaining the muscular work in specific tasks. This information can be used to determine and describe metabolic and/or neuromuscular parameters responsible for task failure and performance reductions typical of fatigue [27]. The validity of the wiNIREM wearable solution suggests the possibility of using this technology to describe and quantify the neuromuscular and physiological demands during different type of exercises (i.e., strength or endurance), providing a comprehensive assessment of muscle function in vivo. Therefore, it is feasible to suggest that the biofeedback provided by these integrated bio-signals could be used to define the individual dose (quantity and intensity of training load in each subject) of training prescription in order to induce specific adaptative responses as well as assess the effectiveness of exercise programs. This application would find merit not only in athletic populations, but also in rehabilitation settings.

We are unable to predict the future research directions of the described muscle activity textile-wearable solution or similar equipment, integrating EMG/NIRS-oximetry, in medicine and sport sciences. On November 2019, nevertheless, the European Space Agency (ESA) signed a 3-year contract with Ohmatex ApS for the development of training tights with integrated EMG/NIRS sensors and wearable computing that should help astronauts on the International Space Station (ISS) to train effectively to maintain their muscle strength and prevent undesirable effects caused by weightlessness [28]. The training tights will also include stretch sensors to track changes in leg volume resulting from fluid displacement or muscle atrophy.

### 3.5. Limitations

The strengths, limitations, and some of the potential confounding factors that can affect the quality and reproducibility of muscle oximetry data were extensively discussed, and recommendations were provided [7]. One of the main limitations of the present study is related to the muscle crosstalk potentially captured by the textile electrodes. In fact, due to the difference in surface areas covered by the standard electrode vs. the textile electrodes (the textile being larger), it is possible that a larger surface area could have been affected more by crosstalk of knee extensor muscles, as previously highlighted [29,30]. Then, the latter could cause a difference in amplitude. Therefore, the findings of this study should be accepted in the context of the signal detection devices used, and not extended to other wearable solutions. Another limitation of the present study is due to the RMS averaging procedure among the four tested running speeds, which did not consider the number of steps performed as a consequence of the running speed and is representative only for the number of steps performed at each speed in the analyzed epochs.

Experience from this study indicates that EMG electrodes integrated in tights use the normal skin perspiration far better than sleeves. Even though the sleeve is tightened, this may be due to a better fit all around the leg. However, various integration design to improve the skin/electrode interface during long term low activity should be tested.

Although the use of the textile EMG is the explicit strength of this study, several factors still need to be considered when measuring EMG activity. To minimize the effects of inter-individual differences in subcutaneous tissue and muscle properties on the signal quality, the results are typically presented as a fraction of the maximal EMG measured during MIVC. Thus, EMG normalized to MIVC represents an effort relative to a muscle group’s force production capacity. wiNIREM allows measurements on thigh and calf muscle regions, although other additional activated muscle groups would need to be monitored. 

Further studies will be necessary to assess the validity and reliability of the data collected on the quadriceps muscle in the finalized garment, as well as its reliability and durability after its prolonged use. This proof of concept study suggests the potential for such design to be developed further.

## 4. Conclusions

The results of this preliminary study indicate that the new wearable solution embedding NIRS and EMG sensing is valid. In fact, the results of the comparison between the regional muscle O_2_Hb saturation and surface EMG measurements (performed either under resting or dynamic conditions by a new wearable integrated NIRS-oximetry/EMG system adopting smart textiles), with those performed using two “gold standard” commercial instrumentations for EMG and muscle oximetry, support the reliability of the data provided by the combined fabric-based wearable EMG sensors and tissue oximetry.

## References

[1] Park Y.-G., Lee S., Park J.-U. (2019). Recent Progress in Wireless Sensors for Wearable Electronics. Sensors.

[2] Cardinale M., Varley M.C. (2017). Wearable Training-Monitoring Technology: Applications, Challenges, and Opportunities. Int. J. Sports Physiol. Perform..

[3] Seshadri D.R., Li R.T., Voos J.E., Rowbottom J.R., Alfes C.M., Zorman C.A., Drummond C.K. (2019). Wearable sensors for monitoring the physiological and biochemical profile of the athlete. Npj. Digit. Med..

[4] Fernández-Caramés T., Fraga-Lamas P. (2018). Towards the Internet-of-Smart-Clothing: A Review on IoT Wearables and Garments for Creating Intelligent Connected E-Textiles. Electronics.

[5] Jouffroy R., Jost D., Prunet B. (2020). Prehospital pulse oximetry: A red flag for early detection of silent hypoxemia in COVID-19 patients. Crit. Care.

[6] UK National Health Service NHS Guidance for Pulse Oximetry to Detect Early Deterioration of Patients with COVID-19 in Primary and Community Care Settings. https://www.england.nhs.uk/coronavirus/publication/pulse-oximetry-to-detect-early-deterioration-of-patients-with-covid-19-in-primary-and-community-care-settings/.

[7] Barstow T.J. (2019). Understanding near infrared spectroscopy and its application to skeletal muscle research. J. Appl. Physiol..

[8] Grassi B., Quaresima V. (2016). Near-infrared spectroscopy and skeletal muscle oxidative function in vivo in health and disease: A review from an exercise physiology perspective. J. Biomed. Opt..

[9] Perrey S., Ferrari M. (2018). Muscle Oximetry in Sports Science: A Systematic Review. Sports Med..

[10] Yoshitake Y., Ue H., Miyazaki M., Moritani T. (2001). Assessment of lower-back muscle fatigue using electromyography, mechanomyography, and near-infrared spectroscopy. Eur. J. Appl. Physiol..

[11] Scano A., Zanoletti M., Pirovano I., Spinelli L., Contini D., Torricelli A., Re R. (2019). NIRS-EMG for Clinical Applications: A Systematic Review. Appl. Sci..

[12] Guo W., Yao P., Sheng X., Liu H., Zhu X. A wireless wearable sEMG and NIRS acquisition system for an enhanced human-computer interface. Proceedings of the Conference Proceedings—IEEE International Conference on Systems, Man and Cybernetics.

[13] Hu G., Zhang Q., Ivkovic V., Strangman G.E. (2016). Ambulatory diffuse optical tomography and multimodality physiological monitoring system for muscle and exercise applications. J. Biomed. Opt..

[14] Kauppi K., Korhonen V., Ferdinando H., Kallio M., Myllylä T. (2017). Combined surface electromyography, near-infrared spectroscopy and acceleration recordings of muscle contraction: The effect of motion. J. Innov. Opt. Health Sci..

[15] Kimoto A., Yamada Y. (2015). A new layered sensor for simultaneous measurement of EMG, MMG and oxygen consumption at the same position. Med. Biol. Eng. Comput..

[16] Ding X., Wang M., Guo W., Sheng X., Zhu X. Hybrid sEMG, NIRS and MMG Sensor System. Proceedings of the Conference: 2018 25th International Conference on Mechatronics and Machine Vision in Practice (M2VIP).

[17] Finni T., Hu M., Kettunen P., Vilavuo T., Cheng S. (2007). Measurement of EMG activity with textile electrodes embedded into clothing. Physiol. Meas..

[18] Colyer S.L., McGuigan P.M. (2018). Textile Electrodes Embedded in Clothing: A Practical Alternative to Traditional Surface Electromyography when Assessing Muscle Excitation during Functional Movements. J. Sports Sci. Med..

[19] Di Giminiani R., Lancia S., Ferrari M., Quaresima V., Vistisen H.T., Kliltgaard A., Heick R.A., Oestergard K., Soerensen K.Y., Cardinale M. A wearable integrated textile EMG and muscle oximetry system for monitoring exercise-induced effects: A feasibility study. Proceedings of the 2018 IEEE International Symposium on Medical Measurements and Applications (MeMeA).

[20] Löfhede J., Seoane F., Thordstein M. (2012). Textile Electrodes for EEG Recording—A Pilot Study. Sensors.

[21] Cömert A., Hyttinen J. (2015). Investigating the possible effect of electrode support structure on motion artifact in wearable bioelectric signal monitoring. Bio. Med. Eng. OnLine.

[22] Grassi B., Porcelli S., Marzorati M. (2019). Translational Medicine: Exercise Physiology Applied to Metabolic Myopathies. Med. Sci. Sports Exercise.

[23] Alkner B.A., Tesch P.A. (2004). Efficacy of a gravity-independent resistance exercise device as a countermeasure to muscle atrophy during 29-day bed rest. Acta Physiol. Scand..

[24] Hermens H.J., Freriks B., Disselhorst-Klug C., Rau G. (2000). Development of recommendations for SEMG sensors and sensor placement procedures. J. Electromyogr. Kinesiol..

[25] Martin B.J., Altman D.G. (1986). Statistical Methods for Assessing Agreement between Two Methods of Clinical Masurement. Lancet.

[26] McManus C.J., Collison J., Cooper C.E. (2018). Performance comparison of the MOXY and PortaMon near-infrared spectroscopy muscle oximeters at rest and during exercise. J. Biomed. Opt..

[27] De Luca C.J. (1997). The Use of Surface Electromyography in Biomechanics. J. Appl. Biomech..

[28] Hackney K.J., Scott J.M., Hanson A.M., English K.L., Downs M.E., Ploutz-Snyder L.L. (2015). The Astronaut-Athlete: Optimizing Human Performance in Space. J. Strength Cond. Res..

[29] Farina D., Merletti R., Indino B., Nazzaro M., Pozzo M. (2002). Surface EMG crosstalk between knee extensor muscles: Experimental and model results. Muscle Nerve.

[30] Talib I., Sundaraj K., Lam C.K., Hussain J., Ali M.A. (2019). A review on crosstalk in myographic signals. Eur. J. Appl. Physiol..

